# In vivo assessment of glutamine anaplerosis into the TCA cycle in human pre-malignant and malignant clonal plasma cells

**DOI:** 10.1186/s40170-020-00235-4

**Published:** 2020-12-11

**Authors:** Wilson I. Gonsalves, Jin Sung Jang, Erik Jessen, Taro Hitosugi, Laura A. Evans, Dragan Jevremovic, Xuan-Mai Pettersson, Alexander Graham Bush, Jaimee Gransee, Emilie I. Anderson, Shaji K. Kumar, K. Sreekumaran Nair

**Affiliations:** 1 Division of Hematology, Mayo Clinic, 200 First Street SW, Rochester, MN 55905 USA; 2 Department of Laboratory Medicine and Pathology, Mayo Clinic, Rochester, MN USA; 3 Department of Health Service Research, Mayo Clinic, Rochester, MN USA; 4 Department of Oncology, Mayo Clinic, Rochester, MN USA; 5 Mayo Clinic Metabolomics Core, Mayo Clinic, Rochester, MN USA; 6 Division of Endocrinology, Mayo Clinic, Rochester, MN USA

**Keywords:** Stable isotope metabolomics, Plasma cell malignancies, Myeloma, Glutamine

## Abstract

**Background:**

Overexpression of c-Myc is required for the progression of pre-malignant plasma cells in monoclonal gammopathy of undetermined significance (MGUS) to malignant plasma cells in multiple myeloma (MM). c-Myc also increases glutamine anaplerosis into the tricarboxylic acid (TCA) cycle within cancer cells. Whether increased glutamine anaplerosis is associated with the progression of pre-malignant to malignant plasma cells is unknown.

**Methods:**

Human volunteers (*N* = 7) and patients with MGUS (*N* = 11) and MM (*N* = 12) were prospectively recruited to undergo an intravenous infusion of ^13^C-labeled glutamine followed by a bone marrow aspiration to obtain bone marrow cells and plasma.

**Results:**

Despite notable heterogeneity, stable isotope-resolved metabolomics (SIRM) revealed that the mean ^13^C-labeled glutamine anaplerosis into the TCA cycle was higher in malignant compared to pre-malignant bone marrow plasma cells relative to the remainder of their paired bone marrow mononuclear cells. RNA sequencing demonstrated a higher relative mRNA expression of c-Myc and glutamine transporters such as ASCT2 and SN2 in malignant compared to pre-malignant bone marrow plasma cells. Finally, higher quantitative levels of TCA cycle intermediates in the bone marrow plasma differentiated MM from MGUS patients.

**Conclusion:**

Measurement of the in vivo activity of glutamine anaplerosis into the TCA cycle provides novel insight into the metabolic changes associated with the transformation of pre-malignant plasma cells in MGUS to malignant plasma cells in MM.

**Trial registration:**

NCT03384108 and NCT03119883

## Key points

- Glutamine anaplerosis into the TCA cycle is relatively higher in malignant bone marrow plasma cells compared to pre-malignant plasma cells.
- Quantitative levels of TCA cycle intermediates differentiate between MGUS and MM bone marrow microenvironments while reflecting their clonal plasma cell burden.

## Introduction

Multiple myeloma (MM) is the second most common hematological malignancy in the USA with over 20,000 new MM patients being diagnosed each year [1]. This malignant plasma cell disorder is associated with devastating end-organ damage such as bone destruction, renal failure, anemia, and hypercalcemia [2]. Despite improvements in survival with the advent of novel therapies [3–5], MM remains mostly incurable with a median survival of only 5 to 7 years [6]. Thus, identifying novel targets in clonal plasma cells (cPCs) from MM that can be exploited for clinical purposes either for early diagnosis or therapeutic purposes remain critical.

Malignant cells have altered cellular metabolism in order to meet increased requirements for nutrients and energy [7]. Although upregulation of aerobic glycolysis (the “Warburg effect”) is common in most cancer cells, it is insufficient to support their increased anabolic metabolism [8]. The energy/nutrient deficit is overcome mostly by the tricarboxylic acid (TCA) cycle, whereby TCA intermediates serve as pre-cursors for the biosynthesis of fatty acids, nucleic acids, and proteins and need constant replenishment [9]. Glutamine anaplerosis or entry into the TCA cycle [10] via the formation of glutamate from glutamine and the subsequent formation of alpha-ketoglutarate from glutamate is one method of replenishing the TCA cycle intermediates [11].

Overexpression of the transcription factor c-Myc is required for the pathogenesis of malignant plasma cells in MM from their pre-cursor state of pre-malignant plasma cells in monoclonal gammopathy of undetermined significance (MGUS). *c-Myc* activation signatures are mostly absent in pre-malignant plasma cells derived from patients with MGUS, whereas 70% of new MM patients have upregulated *c-Myc* activation signatures in their malignant plasma cells [12]. Furthermore, *Myc* activation in germinal center B cells leads to formation of sporadic MM tumors in Vk*MYC mice suggesting its critical role for the progression into MM [13]. Interestingly, c-Myc also promotes transcription of glutamine transporters ASCT2 and SN2 on cell membranes [14] and upregulates GLS1 protein expression to increase conversion of glutamine to glutamate by suppressing mIR23a/b (both being negative regulators of GLS) [15]. Thus, c-Myc leads to increased uptake of glutamine in cells and also increases the conversion of glutamine into glutamate which then enters into the TCA cycle (i.e., glutamine anaplerosis) in mitochondria [16]. However, it remains to be determined whether the level of glutamine anaplerosis into the TCA cycle is higher in malignant plasma cells of the bone marrows of MM patients relative to their adjacent bone marrow mononuclear cells is unknown. And if so, it is uncertain if increasing glutamine anaplerosis activity into the TCA cycle is associated with the progression of pre-malignant plasma cells in MGUS to malignant plasma cells in MM.

There is a gap in knowledge of the activity of metabolic pathways in pre-malignant plasma cells from MGUS due to the lack of adequate in vitro models, as human myeloma cell lines (HMCLs) only represent malignant plasma cells from patients with end-stage MM. In addition, cell culture artifacts from in vitro approaches remain a major barrier in the study of tumor metabolism in humans [17]. To overcome these barriers, this study compared the activity of glutamine anaplerosis into the TCA cycle in pre-malignant plasma cells and malignant plasma cells obtained from MGUS and MM patients, respectively, to their paired remainder of the bone marrow mononuclear cells by using in vivo stable isotope-resolved metabolomics (SIRM) to trace the incorporation of ^13^C glutamine into the intermediates of the TCA cycle.

## Results

### Glutamine anaplerosis into the TCA cycle is present in human myeloma cell lines

The HMCLs, RPMI-8226, and MM1S, like most cancer cell lines, require glutamine supplementation in the cell culture media for optimal proliferation and survival. Our prior study demonstrated glutamine as being the preferred supplier of carbon substrate for the TCA cycle intermediates via its anaplerosis into the TCA cycle in comparison to glucose oxidation when assessed over a time period of 12 h [18]. The expected isotopomer labeling pattern of TCA cycle intermediates when [^13^C_5_]-glutamine undergoes anaplerosis into the TCA cycle is depicted in Supplementary Figure 1. However, this incorporation of ^13^C into the TCA cycle intermediates in HMCLs is rapid as was observed when RPMI-8226 and MM1S HMCLs were cultured for only 60 min in RPMI-1640 media containing 2 mM of glutamine of which only 5% was enriched with [^13^C_5_]-glutamine (Supplementary Figure 2). This rapid uptake of glutamine is not unexpected given the very high proliferation and short doubling time of HMCLs that require nutrients like glutamine for biomass, energy, and redox homeostasis. However, recent studies have demonstrated that the extent of a cancer cell line’s dependence on glutamine anaplerosis may differ based on its environmental context such as the level of cystine [19]. Thus, whether our in vitro observation of glutamine anaplerosis into the TCA cycle of HMCLs is observed in humans in vivo in bone marrow pre-malignant and malignant plasma cells and their paired remainder of the bone marrow mononuclear cells is unclear.

### Intravenous infusion of ^13^C-labeled glutamine in human volunteers is feasible, safe, and subsequently detectable in non-malignant bone marrow plasma cells

**Fig. 1:**
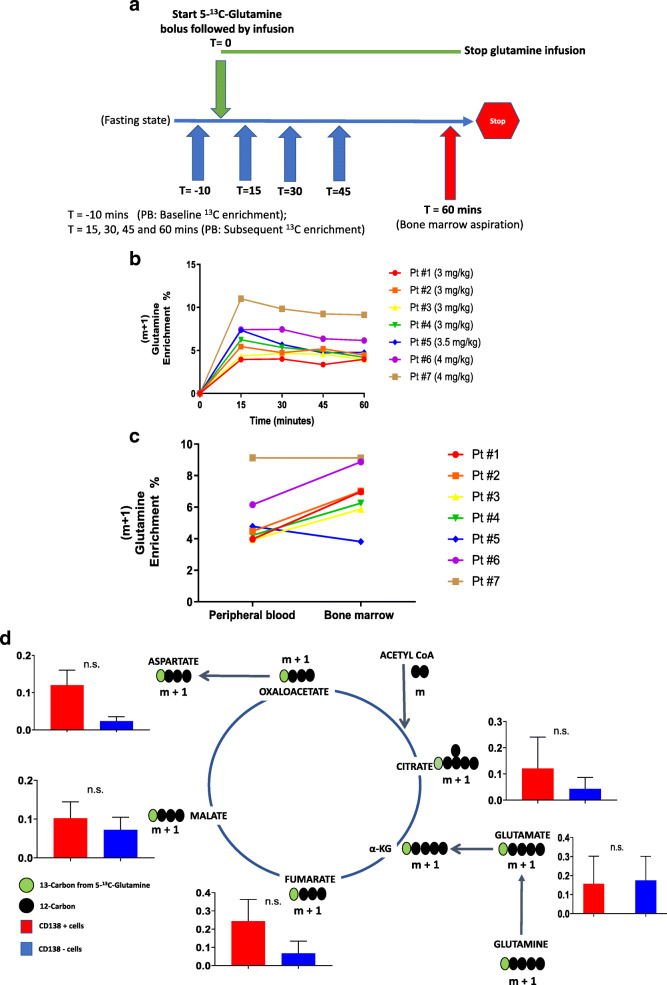
**a** Timeline of procedures/evaluations performed on the volunteers during the study day. **b** Plasma glutamine enrichment during the 5-^13^C-glutamine infusion in each of the volunteers (*N* = 7). **c** Glutamine enrichment in paired bone marrow and peripheral blood plasma after 60 min of the 5-^13^C-glutamine infusion from each of the volunteers (*N* = 7). **d** Comparison of mean relative ^13^C fractional enrichment of TCA cycle intermediates in CD138+ and CD138- cells from the bone marrows of volunteers (*N* = 3). Error bars represent SEM. n.s. is non-significant by paired *t* test

A total of seven healthy volunteers were prospectively recruited to participate in a pilot study evaluating the feasibility and safety of assessing the in vivo utilization of glutamine by their bone marrow plasma cells. All volunteers, in the fasting state, received an infusion of [5-^13^C]-glutamine as a bolus dose over 5 min followed by a continuous infusion of this agent over 60 min prior to undergoing a bone marrow aspiration to harvest bone marrow plasma cells with the infusion ending immediately thereafter (Fig. 1a). The clinical characteristics of these seven volunteers, the bolus and continuous infusion dosages of 5-^13^C-glutamine infused and their pre- and post-infusion serum ammonia and blood urea nitrogen levels which were measured to assess for metabolic toxicities are listed in Table 1. The infusion generated a persistent (m+1) glutamine enrichment in the peripheral blood ranging from 4 to 10% within the first 15 min of the continuous infusion (Fig. 1b). The 5-^13^C-glutamine enrichment was relatively higher in bone marrow plasma compared to paired peripheral blood plasma after 60 min of the 5-^13^C-glutamine infusion (Fig. 1c). We next performed a comparison of sorted CD138+ (non-malignant plasma cells) and paired CD138- cells (remainder of the bone marrow mononuclear cells) for the enrichment of ^13^C in their intracellular TCA cycle intermediates. The cell sorting process for bone marrow mononuclear cells based on CD138 expression of four of the seven volunteers were inadequate for analysis due to technical processing errors incurred initially in this study. Thus, sorted cells were available only in three volunteers, and the enrichment of ^13^C in their intracellular TCA cycle intermediates was normalized to the steady state ^13^C (m+1) glutamine enrichment in their respective bone marrow plasma samples. Figure 1d shows the observed enrichment of ^13^C in the intracellular intermediates from the first turn of the TCA cycle in CD138+ plasma cells and their paired CD138- bone marrow mononuclear cells. This demonstrated the feasibility of the in vivo SIRM methods for assessing glutamine anaplerosis into the TCA cycle in bone marrow plasma cells and the remainder of their paired bone marrow mononuclear cells.

**Table 1:** Characteristics of the volunteers participating and the dosages of 5-^13^C-glutamine infused and their pre- and post-blood plasma urea nitrogen (BUN) and ammonia levels

Pt #	Age	Sex	Glutamine dose		BUN (mg/dL)		NH_**3**_ (μmol/L)	
			Prime dose	Infusion dose	Pre	Post	Pre	Post
1^a^	35	F	3 mg/kg	3 mg/kg	15	14	< 10	15
2^a^	24	M	3 mg/kg	3 mg/kg	20	15	12	< 10
3^a^	27	F	3 mg/kg	3 mg/kg	16	16	< 10	< 10
4^a^	29	M	3 mg/kg	3 mg/kg	12	12	15	18
5	24	M	3.5 mg/kg	3.5 mg/kg	16	15	< 10	< 10
6	29	F	4 mg/kg	4 mg/kg	19	13	< 10	24
7	52	F	4 mg/kg	4 mg/kg	9	13	< 10	19

^a^The participants whose non-malignant plasma cells from the marrow were unable to undergo isotopomer assessments by GC-MS due to technical complications

### Glutamine anaplerosis into the TCA cycle in pre-malignant and malignant plasma cells relative to their paired bone marrow mononuclear cells

**Fig. 2:**
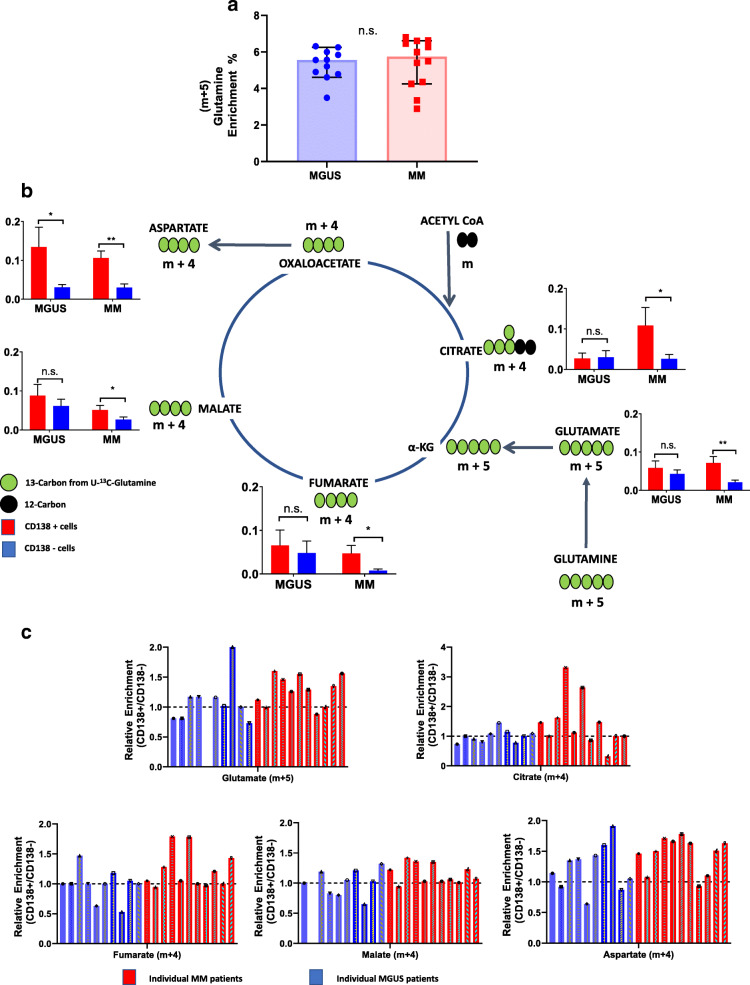
**a** Glutamine enrichment in bone marrow plasma after 60 min of the [^13^C_5_]-glutamine infusion in patients with MGUS (*N* = 11) and MM (*N* = 12). Error bars represent SEM. n.s. is non-significant by unpaired *t* test. **b** Comparison of mean relative ^13^C fractional enrichment of TCA cycle intermediates in CD138+ and CD138- cells from the bone marrows of MGUS (*N* = 10) and MM (*N* = 11). Error bars represent SEM. n.s. is non-significant, *p < 0.05, **p < 0.01, and ***p < 0.001 by paired *t* test. **c** Relative ^13^C fractional enrichment (CD138+/CD138-) of TCA cycle intermediates from each MGUS (*N* = 10) and MM (*N* = 11) patient.

We next evaluated the amount of glutamine anaplerosis into the TCA cycle of pre-malignant and malignant plasma cells from MGUS and MM patients respectively relative to the remainder of their paired bone marrow mononuclear cells. A total of eleven MGUS and twelve MM patients were prospectively enrolled on this study. To improve the mass spectrometry (MS) detection of the various TCA cycle isotopologues of interest, we used an intravenous infusion of [^13^C_5_]-glutamine (i.e., as it would form m+5 and m+4 isotopomers in the first turn of the TCA cycle) instead of the previously utilized 5-^13^C-glutamine (which forms m+1 isotopomers in the first turn of the TCA cycle). [^13^C_5_]-glutamine was administered as a bolus dose (4 mg/kg) over 5 min followed by a continuous infusion (4 mg/kg/h) over 60 min prior to undergoing a bone marrow aspiration to harvest bone marrow plasma cells with the infusion ending immediately thereafter. The clinical characteristics of these MGUS and MM patients and their corresponding bolus and continuous infusion dosages of [^13^C_5_]-glutamine are listed in Tables 2 and 3, respectively. The infusion generated a (m+5) glutamine enrichment ranging from 3 to 7% in the bone marrow plasma after completing 60 min of the continuous infusion which was similar between MGUS and MM patients (Fig. 2a). Upon acquiring the bone marrow aspirate sample, subsequent processing was performed to yield bone marrow CD138+ cells (pre-malignant or malignant plasma cells) and paired CD138- (remainder of the bone marrow mononuclear cells) from each aspirate sample. The cell sorting process for bone marrow mononuclear cells based on CD138 expression yielded sufficient clonal pre-malignant plasma cells in only ten of the eleven MGUS patients and sufficient clonal malignant plasma cells in only eleven of the twelve MM patients. As a result, isotopomer data evaluating the incorporation of ^13^C from glutamine into the TCA cycle intermediates of CD138+ and their paired CD138- cells was available for assessment in only ten MGUS and eleven MM patients (Fig. 2b). The mean ^13^C labeling of the TCA cycle intermediates (glutamate, citrate, fumarate, malate, and aspartate) derived from [^13^C_5_]-glutamine anaplerosis into the first turn of the TCA cycle were higher in CD138+ malignant plasma cells from MM patients compared to the remainder of their paired CD138- mononuclear cells. In contrast, the mean ^13^C labeling of the TCA cycle intermediates derived from [^13^C_5_]-glutamine anaplerosis into the TCA cycle were not significantly higher in CD138+ pre-malignant plasma cells from MGUS patients compared to the remainder of their paired CD138- mononuclear cells except for the metabolite aspartate. To better understand the observed differences in the mean ^13^C labeling of the TCA cycle intermediates between the two disease groups, the ^13^C labeling of individual TCA cycle intermediates were also evaluated in each individual MGUS and MM patient by assessing their fractional ^13^C enrichment (i.e., ratio of ^13^C enrichment of a TCA metabolite in CD138+ cells/CD138- cells such that a ratio of > 1 reflects higher ^13^C labeling of the specific metabolite in CD138+ cells). This analytical approach demonstrated notable variability in the fractional ^13^C enrichment of individual TCA cycle intermediates within pre-malignant and malignant CD138+ plasma cells obtained from MGUS and MM patients respectively (Fig. 2c). Thus, the glutamine anaplerosis into the TCA cycle based on the mean ^13^C labeling of the TCA metabolites was higher in malignant than pre-malignant CD138+ bone marrow plasma cells when compared to the remainder of their paired CD138- bone marrow mononuclear cells. However, this increased glutamine anaplerosis into the TCA cycle in CD138+ malignant plasma cells compared to the remainder of their paired CD138- mononuclear cells was not uniformly observed in all MM patients. On the contrary, it was also observed even in few MGUS patients and their CD138+ pre-malignant plasma cells when compared to the remainder of their paired CD138- mononuclear cells suggesting significant heterogeneity in the activity of this pathway among the pre-malignant and malignant CD138+ plasma cells from MGUS and MM patients, respectively. We do not have sufficient follow-up data to assess whether those MGUS patients who demonstrated increased fractional ^13^C enrichment of the TCA cycle intermediates in their CD138+ pre-malignant plasma cells when compared to the remainder of their paired CD138- mononuclear cells were more likely to develop MM sooner compared to the remainder of the MGUS patients.

**Table 2:** Clinical characteristics of the MGUS patients and their corresponding bolus and continuous infusion dosages of [^13^C_5_]-glutamine

Patient #	Age	Sex	M-Spike	Isotype	Glutamine enrichment
**MGUS #1**^**c**^	52	F	0.4 g/dL	IgG lambda	5.21%
**MGUS #2**^**a**^	65	M	NQ; IgA, 476 mg/dL	IgA kappa	4.78%
**MGUS #3**^**c**^	71	M	1.2 g/dL	IgG lambda	4.61%
**MGUS #4**^**c**^	66	M	0.7 g/dL	IgG kappa	5.57%
**MGUS #5**^**b**^	69	M	1.4 g/dL	IgG lambda	3.49%
**MGUS #6**	48	M	1.2 g/dL	IgG kappa	4.90%
**MGUS #7**	56	F	1.2 g/dL	IgG kappa	5.84%
**MGUS #8**^**c**^	61	F	NQ; IgG, 867 mg/dL	IgG lambda	6.31%
**MGUS #9**^**c**^	71	M	NQ; kappa FLC, 47.5 mg/dL	Free kappa	6.25%
**MGUS #10**	67	F	0.6 g/dL	IgG kappa	5.56%
**MGUS #11**	56	F	NQ; IgA, 464 mg/dL	IgA kappa	6.00%

*NQ* non-quantifiable

^a^The participant whose pre-malignant plasma cells from the marrow were unable to be analyzed for the isotopomers of malate by GC-MS

^b^The participant whose pre-malignant plasma cells from the marrow were unable to be analyzed for the isotopomers of glutamate by GC-MS

^c^The participant who did not have sufficient pre-malignant plasma cells from the marrow to undergo RNA sequencing

**Table 3:** Clinical characteristics of the MM patients and their corresponding bolus and continuous infusion dosages of [^13^C_5_]-glutamine

Patient #	Age	Sex	M-Spike	Isotype	Glutamine enrichment	Primary cytogenetics	Prior lines of therapy	BMPC%	S-Phase%
**MM #1**	72	F	1.3 g/dL	IgG kappa	4.25%	Trisomies	2	20%	1.6%
**MM #2**	65	F	NQ	NS	2.89%	t(11;14), +1q	4	95%	23.8%
**MM #3**	71	M	1.5 g/dL	IgG kappa	5.40%	Trisomies	3	15%	0.7%
**MM #4**	71	M	0.5 g/dL	IgG kappa	5.49%	Trisomies, +1q	1	40%	2.6%
**MM #5**	76	F	NQ; kappa FLC, 47.6 mg/dL	Free kappa	6.61%	Myc sep, +1q, -17p	2	15%	3.0%
**MM #6**	58	F	NQ; lambda FLC, 11 mg/dL	Free lambda	6.80%	IgH sep, -17p	3	100%	8.2%
**MM #7**^**a**^	53	M	1.9 g/dL	IgG kappa	3.35%	N/A	5	0%	N/A
**MM #8**	69	F	NQ; lambda FLC, 51 mg/dL	Free lambda	6.63%	Trisomies, +1q	1	20%	4.5%
**MM #9**	75	M	Lambda FLC, 40.5 mg/dL	Free lambda	6.45%	Trisomies	4	20%	2.0%
**MM #10**	71	M	1.9 g/dL	IgA kappa	6.27%	t(11;14), +1q, -17p	3	50%	1.3%
**MM #11**	71	M	NQ	NS	5.99%	t(11;14), -17p	2	1%	N/A
**MM #12**^**a**^	61	M	1.2 g/dL	IgG kappa	4.35%	Trisomies	1	5%	1.0%

*NQ* non-quantifiable, *NS* non-secretory, *BMPC%* bone marrow plasma cell percentage

^a^Participant who did not have sufficient malignant plasma cells from the marrow to undergo RNA sequencing

### Transcriptomic analysis of the expression of c-Myc and glutamine transporters on cell membranes in pre-malignant or malignant plasma cells and their paired remainder of bone marrow mononuclear cells

**Fig. 3:**
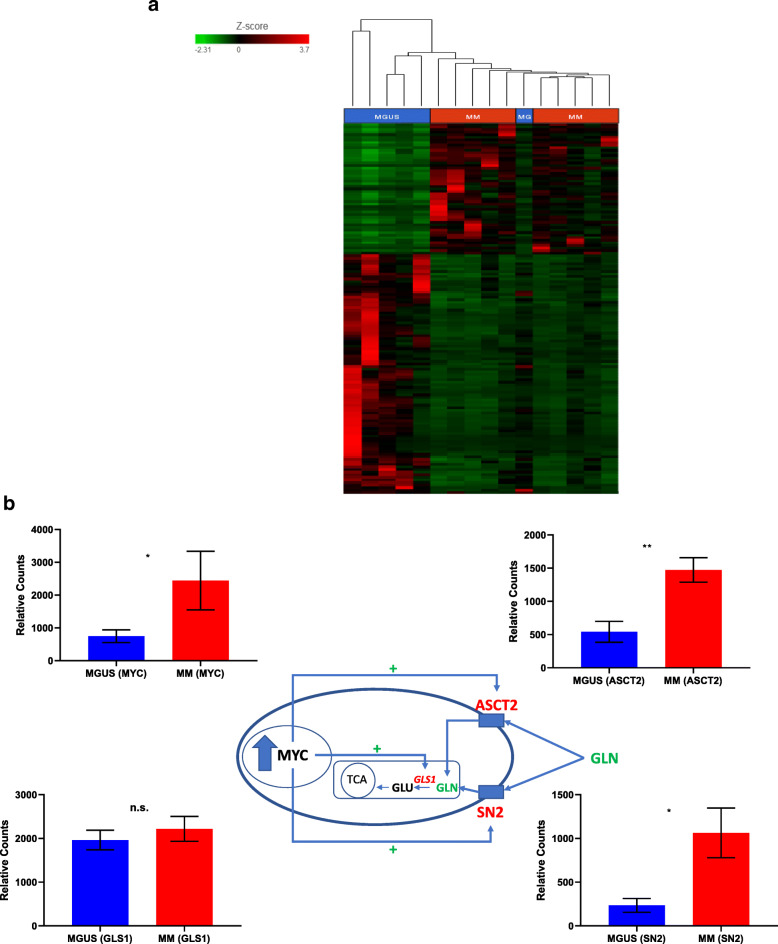
**a** Hierarchical clustering analysis on the overall differential expression of genes between MGUS (*N* = 6) and MM (*N* = 10) patients. **b** Relative differences in mRNA expression of c-Myc, GLS, ASCT2, and SN2 between CD138+ cells from MGUS (*N* = 6) and MM (*N* = 10) patients in context of their effect on glutamine transport into the cell

Transcriptional upregulation of genes involved in glutamine anaplerosis is one of the possible explanations for the differences in glutamine anaplerosis activity to exist between any two groups if present. Our a priori hypothesis was that increased *c-Myc* expression leads to the progression of pre-malignant plasma cells in MGUS to malignant plasma cells in MM with a resultant increase in glutamine anaplerosis into the TCA cycle that is facilitated by the overexpression of GLS1 that converts glutamine to glutamate as well as by overexpression of the glutamine cell membrane transporters ASCT2 and SN2. Therefore, we examined the RNA expression profile of CD138+ pre-malignant and malignant plasma cells as well as their paired CD138- mononuclear cells from the bone marrows of MM and MGUS patients using RNA sequencing. Sufficient RNA was obtained from six patients with MGUS and ten patients with MM. A total of 2300 genes were determined to be differentially expressed between the malignant and pre-malignant CD138+ plasma cells from MM and MGUS groups, respectively, of which 799 genes were overexpressed and 1501 were under expressed. Hierarchical clustering analysis was performed demonstrating the overall differential expression of genes between the two groups (Fig. 3a). As expected, there was an increased relative expression of c-Myc mRNA in CD138+ malignant plasma cells from MM compared to CD138+ pre-malignant plasma cells from MGUS as well as an increase in the mRNA expression of glutamine importers such as ASCT2 and SN2 (Fig. 3b). However, the expression of GLS1 was similar between the CD138+ malignant and pre-malignant plasma cells. Furthermore, the relative mRNA expression of other glutamine transporter such as SNAT1 and LAT1 were not significantly different between the CD138+ malignant and pre-malignant plasma cells (Supplemental Figure 3). There was also an increased relative mRNA expression of c-Myc, GLS1, ASCT2, and SN2 in CD138+ malignant plasma cells compared to their paired CD138- mononuclear cells in MM patients supporting the previous observation of a higher mean ^13^C labeling of the TCA cycle intermediates (glutamate, citrate, fumarate, malate, and aspartate) in CD138+ malignant plasma cells from MM patients compared to their paired CD138- mononuclear cells. Interestingly, the relative mRNA expression of c-Myc, GLS1, and ASCT2 in CD138+ pre-malignant plasma cells was also higher than their paired CD138- mononuclear cells in MGUS patients although not to the same extent as seen in MM patients (Supplementary Table 1). This suggests that even at the MGUS stage, there may be a preferential utilization of glutamine by CD138+ pre-malignant plasma cells over their paired CD138- mononuclear cells. It supports the previous observation of higher fractional ^13^C enrichment of the TCA cycle intermediates in the CD138+ pre-malignant plasma cells from individual MGUS patients compared to their paired CD138- mononuclear cells. However, we cannot adequately verify this hypothesis since we were unable to simultaneously compare the relative mRNA expression of c-Myc, GLS1, and ASCT2 in CD138+ polyclonal plasma cells and their paired CD138- mononuclear cells from the bone marrows aspirates of the healthy donors in this study to determine if it was different than that in MGUS patients.

### Quantitative levels of TCA cycle intermediates in the bone marrow plasma of patients with MGUS and MM

**Fig. 4:**
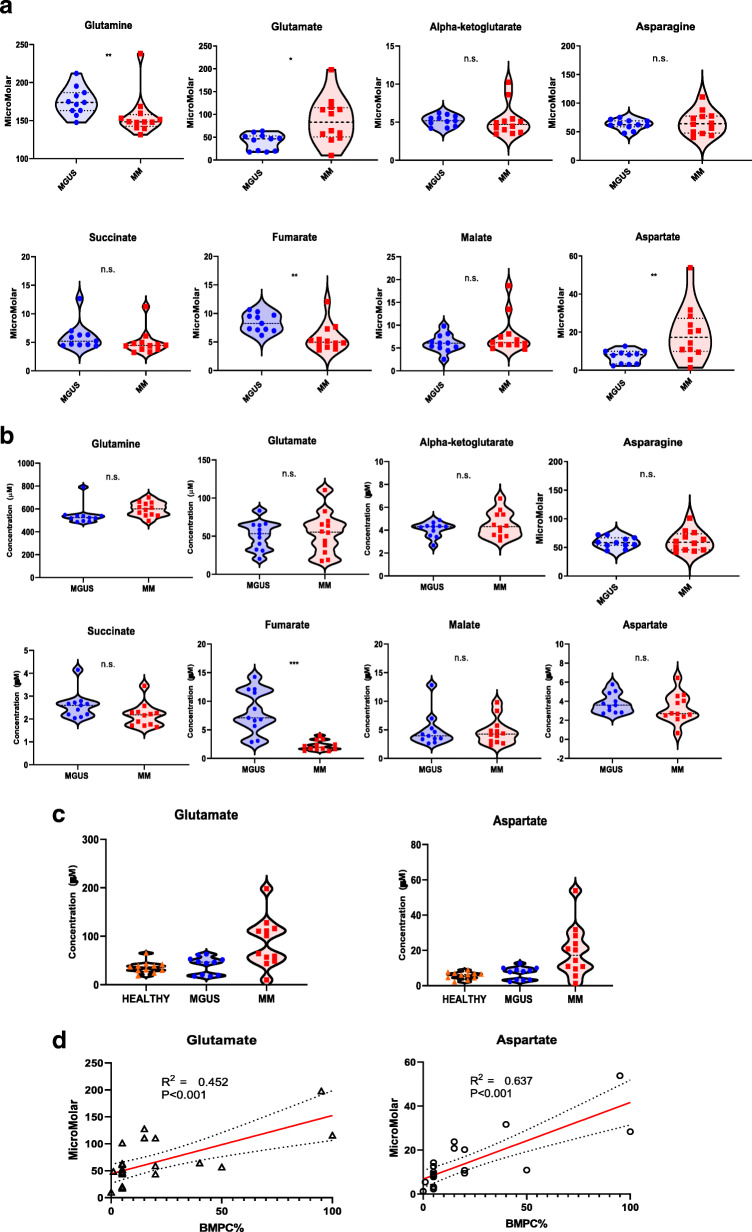
**a** Violin plots comparing the median concentrations of different TCA metabolites in the bone marrow plasma between MGUS (*N* = 11) and MM (*N* = 12) groups. Data was analyzed by Mann-Whitney *U* test where ***p < 0.001, **p < 0.01, *p < 0.05, ^#^*p* < 0.1 but ≥ 0.05, n.s. non-significant. **b** Violin plots comparing the median concentrations of different TCA metabolites in the peripheral blood plasma between MGUS (*N* = 11) and MM (*N* = 12) groups. Data was analyzed by Mann-Whitney *U* test where ***p < 0.001, **p < 0.01, *p < 0.05, ^#^*p* < 0.1 but ≥ 0.05, n.s. non-significant. **c** Violin plots comparing the median concentrations of aspartate and glutamate in the bone marrow plasma between the volunteers (*N* = 7), MGUS (*N* = 11), and MM (*N* = 12) groups. Data was analyzed by Mann-Whitney *U* test where ***p < 0.001, **p < 0.01, *p < 0.05, ^#^*p* < 0.1 but ≥ 0.05, n.s. non-significant. **d** XY-correlation plots comparing the concentrations of aspartate and glutamate in the bone marrow plasma of patients with MGUS and MM and the percentage of clonal plasma cells in their bone marrow

Though heterogenous, since the average in vivo activity of glutamine anaplerosis into the TCA cycle was different between CD138+ malignant plasma cells from MM patients and their paired CD138- bone marrow mononuclear cells, we assessed the absolute quantification of the concentrations of these TCA cycle intermediates in the bone marrow and peripheral blood plasma of these MM patients and compared them to those from the MGUS patients. Interestingly, there were significantly higher levels of glutamate and aspartate and lower levels of glutamine in the bone marrow plasma of MM patients compared to that obtained from MGUS patients (Fig. 4a). In contrast, there were no significant differences in the levels of TCA cycle intermediates in the peripheral blood plasma of MM patients compared to MGUS patients (Fig. 4b). One likely explanation for this difference is that the bone marrow plasma is the closest to representing the surrounding microenvironment of the pre-malignant and malignant CD138+ plasma cells as well as their CD138- mononuclear cells unlike peripheral blood plasma. It is possible that either the intracellular differences in the levels of the TCA cycle intermediates between pre-malignant and malignant bone marrow CD138+ plasma cells or the differences between the CD138- bone marrow mononuclear cells from MGUS and MM patients are more likely to be captured from extracellular assessments in the bone marrow plasma rather than the peripheral blood plasma. To ensure that the different concentrations of TCA metabolites such as glutamate and aspartate noted in the bone marrow plasma samples when compared to their paired peripheral blood plasma samples from the same patient was not due to an unknown technical issue with the GC-MS methodology used, the absolute concentrations in the bone marrow plasma samples of all MGUS and MM patients were verified by liquid chromatography-mass spectrometry (LC-MS) and showed a high correlation (Supplementary Figure 4). Interestingly, in a cross-comparison assessment of the bone marrow plasma concentrations of glutamate and aspartate between the previously described human volunteers and the patients with MGUS and MM, the levels of glutamate and aspartate in MGUS patients was akin to that observed in the human volunteers which together were both lower than that detected in MM patients (Fig. 4c). Furthermore, the levels of both these TCA cycle metabolites directly correlated to the percentage of CD138+ plasma cells present in their bone marrows (Fig. 4d).

**Fig. 5:**
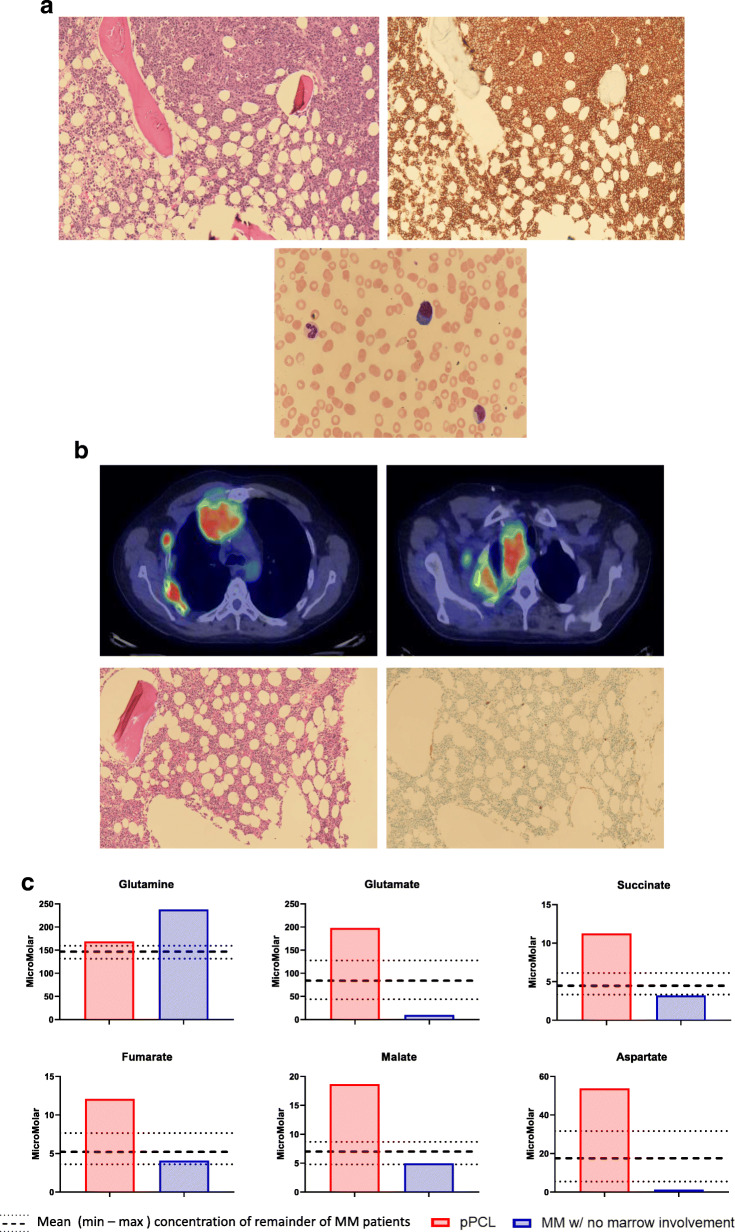
**a** Immunohistochemical staining of the bone marrow for CD138+ clonal plasma cells in MM patient #2 with primary plasma cell leukemia as well as peripheral smear evaluation demonstrating the presence of circulating plasma cells. **b** Positron emission tomography-computed tomography (PET*/*CT) demonstrating the presence of a large plasmacytoma in the right chest wall as well as immunohistochemical staining of the bone marrow for CD138+ clonal plasma cells in MM patient #7 with no marrow involvement. **c** Graphical visualization of the concentrations of the TCA cycle intermediate such as glutamate, succinate, fumarate, malate, and aspartate in the bone marrow plasma of MM patient #2 with primary plasma cell leukemia and MM patient #7 with respect to that of the remainder of the MM patients (represented by the mean (dashed line) and maximum and minimum (dotted lines) values)

Finally, among the MM patients enrolled on this study, there were two patients (MM #2 and MM #7) who had distinct clinical phenotypes. The first patient (MM #2) had relapsed/refractory primary plasma cell leukemia with an extensive amount of bone marrow infiltration with malignant plasma cells as well as a high number of circulating malignant plasma cells (Fig. 5a). The second patient (MM #7) had relapsed/refractory MM but all the malignant plasma cells were confined to his chest and mediastinum with no bone marrow involvement in the posterior iliac crest since diagnosis typically seen with the other MM patients (Fig. 5b). The concentrations of the TCA cycle intermediate such as glutamate, succinate, fumarate, malate, and aspartate were markedly higher in the bone marrow plasma of MM #2 compared to the remainder of the MM patients likely reflecting the extensive malignant plasma cell burden in the posterior iliac crest site of the bone marrow aspiration (Fig. 5c). In contrast, the concentrations of the TCA cycle intermediate such as glutamate, succinate, and aspartate were markedly lower and the concentration of glutamine was markedly higher in the bone marrow plasma of MM #7 compared to the remainder of the MM patients likely reflecting the absence of any malignant plasma cells in the posterior iliac crest site of the bone marrow aspiration.

## Discussion

The current study demonstrated the in vivo activity of glutamine anaplerosis into the TCA cycle in pre-malignant and malignant bone marrow plasma cells from patients with MGUS and MM, respectively. Here, we show that evaluating the differences between the intracellular metabolism of malignant plasma cells and their paired non-malignant mononuclear cells in the bone marrow is feasible and can offer valuable insight into the metabolic transformation of pre-malignant plasma cells in MGUS to malignant plasma cells in MM. Furthermore, this study also demonstrated that the concentrations of TCA cycle metabolites in the extracellular space such as the bone marrow plasma which is akin to the microenvironment reflect the differences between the intracellular metabolism of malignant and pre-malignant plasma cells or possibly even between the remainder of their paired CD138- mononuclear cells.

While the glutamine anaplerosis into the TCA cycle based on the mean ^13^C labeling of the TCA metabolites was higher in malignant compared to pre-malignant CD138+ bone marrow plasma cells relative to the remainder of their paired CD138- bone marrow mononuclear cells (Fig. 2b), this was heterogeneously observed among individual MGUS and MM patients (Fig. 2c). There were some MGUS patients whose CD138+ pre-malignant plasma cells had higher fractional ^13^C labeling of their TCA metabolites when compared to the remainder of their paired CD138- mononuclear cells. It would be intriguing if these aforementioned MGUS patients would have a shorter time to progression to MM; however, the study follow-up period is not currently sufficient enough to assess this possibility. Nevertheless, this in vivo SIRM approach to assess the activity of other metabolic pathways of interest in addition to glutamine anaplerosis in bone marrow plasma cells remains promising. This is even more applicable in smoldering multiple myeloma (SMM), a more advanced, asymptomatic clonal plasma cell disorder associated with a higher tumor burden than MGUS, but without the end-organ damage seen in MM [20]. Unlike MGUS, where the risk of progression to MM is 1% per year, the risk of progression in SMM is heterogeneous and decreases with time (~ 10%/year for the first 5 years, ~ 3%/year for the next 5 years, and ~ 1%/year thereafter) [21]. This suggests that SMM is a mix of patients with pre-malignant plasma cells as in MGUS and patients with malignant plasma cells as in MM but who have not yet developed end-organ damage. Some patients with SMM rapidly progress to MM within 1 to 2 years of their diagnosis, suggesting that their plasma cells were already malignant and had biologically progressed to MM [22]. However, at diagnosis, the stratification of SMM patients is not obvious given the lack of end-organ damage (e.g., bone destruction or renal failure), and no reliable clinical or pathological methods are available to differentiate between pre-malignant plasma cells in MGUS and malignant plasma cells in MM. Adding ^13^C SIRM profiling of intracellular metabolic pathways to assist in differentiating pre-malignant from malignant plasma cells could bridge this gap and enable early identification of SMM patients at high risk of progression. Clinical benefit of early therapy in SMM patients likely to progress is well documented [23]. Conversely, identification of those SMM patients at low risk of progression to MM reduces anxiety and prevents risk of toxicity from unnecessary intervention.

While not unexpected, it was interesting to observe that the relative ^13^C labeling of the intermediates from the first turn of the TCA cycle in RPMI-8226 and MM1S HMCLs when cultured in media containing 5% enrichment with [^13^C_5_]-glutamine for 60 min (Supplementary Figure 2) was higher than observed in the CD138+ malignant plasma cells from MM patients who had similar levels of [^13^C_5_]-glutamine enrichment in their bone marrow plasma for approximately the same duration (Fig. 2b). The very high proliferation and short doubling time of HMCLs compared to most primary CD138+ malignant plasma cells from MM patients is likely one explanation for this difference as HMCLs are typically reflective of disease biology that is most aggressive and derived from MM patients at the end stages of their disease. However, another explanation could be that the differences observed in the extent of glutamine utilization between HMCLs and primary CD138+ malignant plasma cells from MM patients may be based on its environmental context such as ex vivo versus in vivo artifacts assessments [19]. The concentrations of nutrients in cell culture media are not reflective of the true physiological levels experienced by primary CD138+ malignant plasma cells from MM patients [17]. This has major implications when evaluating the potential efficacy of metabolic inhibitors in the pre-clinical setting.

The overexpression of glutamine transporters such as ASCT2 and SN2 observed in malignant CD138+ cells compared to the remainder of their paired bone marrow CD138- mononuclear cells point to their potential as therapeutic targets against glutamine metabolism that are worth investigating in the future in MM models. In fact, Bolzoni et al. previously demonstrated the high expression of such glutamine transporters and inhibition of ASCT2 could hinder MM growth in both in vitro and in vivo models [24]. It would be even more intriguing if these transporters could be modulated to prevent the progression of pre-cursor conditions like MGUS to MM. Finally, it was interesting to observe that the mean ^13^C labeling of aspartate derived from [^13^C_5_]-glutamine anaplerosis into the TCA cycle was significantly higher in both CD138+ pre-malignant and malignant plasma cells from MGUS and MM patients, respectively, when compared to the remainder of their paired CD138- mononuclear cells. Though not statistically significant, this increased mean ^13^C labeling of aspartate in the CD138+ cells derived from 5-^13^C-glutamine was also observed in the volunteer cohort. This supports prior observations of increased glutamine utilization by the TCA cycle to form aspartate in normal human polyclonal plasma cells and is thought to be important in supporting their function of antibody production [25]. Thus, it is likely that since both pre-malignant and malignant CD138+ plasma cells are constantly producing excess monoclonal antibodies, their utilization of glutamine for aspartate production is equally higher than the remainder of their paired CD138- mononuclear cells.

The concentrations of several TCA metabolites especially glutamate and aspartate reflected the disease status (MGUS vs. MM as seen in Fig. 4a) and degree of bone marrow clonal plasma cell involvement (Fig. 4d). As a result, we were able to observe two extreme clinical phenotypes among MM patients (MM #2 vs. MM #7) reflected in the differences between their concentrations of certain TCA metabolites in the bone marrow plasma. Interestingly, MM #2 had the lowest (m+5) glutamine enrichment in the bone marrow plasma at ~ 2.85% compared to the remainder of the MM patients despite receiving the same weight-based dose of the stable isotope infusion. A possible explanation could be that the large number of malignant plasma cells consumed most of the intravenously infused U-^13^C-glutamine, thus preventing the bone marrow plasma enrichment from getting to higher level as seen with most of the other MM patients. MM #7 also had the second lowest (m+5) glutamine enrichment in the bone marrow plasma after MM #2 at ~ 3.35% compared to the remainder of the MM patients. Again, a possible explanation could be that the presence of several large plasmacytomas comprised of malignant plasma cells in the chest consumed a lot of the intravenously infused U-^13^C-glutamine preventing the bone marrow plasma enrichment from getting to a higher level. However, these explanations remain speculative and cannot be confirmed in this study. It is possible that other patient or disease-related factors are responsible for these observation differences in the (m+5) glutamine enrichment in the bone marrow plasma among the MM patients.

There are several limitations to this human study. First, it is not known if isotopic steady state was reached in the fraction of CD138+ and CD138- cells from the bone marrow as this would require multiple sampling of bone marrow during the infusion which was not feasible. However, we not only sample similar time points following isotope infusion but also the analytical approach we used here does not require isotopic steady state as we determined the pre-cursor product relationship by normalizing isotopic enrichment of the various citric acid cycle metabolites to plasma glutamine enrichment. Second, there is likely significant attrition of the ^13^C labeling within the fraction of CD138+ and CD138- cells from the bone marrow sample as it undergoes various cell sorting processes. However, it is likely that the tracer and tracee loss occurred similarly such that the enrichment values are indicative of the incorporation of glutamine (via glutamate) into the TCA cycle pathway. Moreover, the normalization procedure of the ^13^C labeling fractions to the enrichment of the stable isotope in the bone marrow plasma as well as relative comparisons of the CD138+ fractions to their paired CD138- fractions allow for adequate comparisons between different groups like MGUS and MM. Third, the healthy participants who enrolled in this study were considerably younger in age (median, 29 years) compared to those patients in the MGUS (median, 65 years) and MM (median, 71 years) groups. This is partly due to the typical healthy donor participant pool consisting mostly of younger individuals. While the direct impact of chronologic age on the degree of glutamine uptake and anaplerosis into the TCA cycle of bone marrow cells has not been ascertained, it is a potential confounder that requires further investigation. Despite these limitations, this study demonstrates the feasibility of gaining insight into the metabolic underpinnings of MM by evaluating the activity of intracellular metabolic networks in malignant plasma cells and their pre-malignant pre-cursors in the bone marrow via in vivo stable isotope tracing methodologies. Similar human studies utilizing SIRM methods have been performed in various cancers like lung cancer [26–29], kidney cancer [30], and glioblastoma [31] utilizing a variety of different stable isotope tracers to assess intracellular metabolic pathways.

## Conclusion

Measurement of the in vivo activity of glutamine anaplerosis into the TCA cycle in bone marrow plasma cells is feasible. It provides novel insight into the metabolic changes associated with the transformation of pre-malignant plasma cells in MGUS to malignant plasma cells in MM. In the future, larger studies may not only be undertaken to assess the in vivo measurement of glutamine anaplerosis or even other metabolic pathways as a marker of conversion of MGUS or SMM to MM but also to assess potential effects of molecules that promise to arrest the progression of MGUS or SMM to MM.

## Methods

### Research protocol for bone marrow assessment of human volunteers and patients with PC disorders

All methods carried out in this study were in accordance with relevant guidelines and regulations. Approval for this study (IRB# 17-00313) was obtained from the Mayo Clinic IRB in accordance with the federal regulations and the principles of the Declaration of Helsinki. Prospective human volunteers were recruited following local advertisements under the clinical trial NCT03384108, whereas consecutive patients with MM and MGUS were prospectively recruited in the clinic under the clinical trial NCT03119883. The diagnostic criteria of International Myeloma Working Group (IMWG) were applied to confirm the diagnosis of MGUS and MM. Upon accrual on either study, participants underwent admission to the clinical research and translational unit for performance of the study procedures. The study procedures involved peripheral blood collections at baseline. The next step was to undergo a bolus injection followed by a continuous infusion of either 5-^13^C-glutamine (3–4 mg/kg bolus and 3–4 mg/kg/h continuous infusion) or [^13^C_5_]-glutamine (4 mg/kg bolus and 4 mg/kg/h continuous infusion) in the volunteers and MGUS/MM patients, respectively. Serial peripheral blood assessments were performed only in the volunteers every 15 min upon the start of the continuous infusion to assess the enrichment of (m+1) glutamine in the peripheral blood. After 60 min of receiving the ^13^C-glutamine infusions, a bone marrow aspiration was performed on the posterior iliac crest and whole bone marrow aspirate samples were collected in cold EDTA tubes for immediate processing.

### Extraction of CD138+ and CD138- mononuclear cells

The freshly obtained bone marrow aspirates from patient underwent Ficoll-Paque gradient separation for plasma processing which was stored for later analysis at − 80 °C. The remnant cellular component of the bone marrow aspirate underwent red cell lysis using ACK lysis buffer. The clonal PCs were extracted using positive selection by mixing the cells with a CD138 positive selection cocktail and anti-CD138 magnetic-activated cell separation microbeads (ROBOSEP™ cell separation system, StemCell Technologies Inc.) in an automated RoboSep cell separation system. Purity of the sorted clonal PCs was confirmed via light chain restriction using slide-based immunofluorescent method.

### RNA-seq and data analysis

Total RNA was extracted from cells and the extracted RNAs were evaluated using a Qubit (Thermo Fisher Scientific, MA, USA) and Agilent 2100 Bioanalyzer (Agilent Technologies, Santa Clara, CA, USA), respectively. The TrueSeq RNA Exome Kit (Illumina, CA, USA) was used to generate the mRNA-seq library according to the manufacturer’s protocol. Constructed libraries were quantified by Bioanalyzer 2100 system using the D1000 kit (Agilent) and Qubit dsDNA BR Assay kits (Thermo Fisher Scientific). All libraries were pooled and sequenced 101 bp paired-end reads on Illumina HiSeq 4000. FASTQ files were uploaded into Partek Flow software (Partek Inc., MO, USA), and primary QC was performed. The STAR (2.7.3a) aligner was used to align reads to the human reference genome (hg38). After alignment, the final BAM files were quantified using Partek E/M algorithm [32] by Ensembl annotations (Ensembl Transcripts release 92). DESeq2 package [33] was used to normalize data and determine differential expression of RNA-seq between the groups. The false discovery rate (FDR) by Benjamini and Hochberg method was used to adjust for comparison. FDR value less than 0.05 and fold change ± 2 at raw *p* value less than 0.05 were considered a significant change. Hierarchical clustering was performed on the significantly changed genes based on the Euclidean distance and the average linkage clustering algorithm using Partek Flow software.

### Methodology for quantitative assessments of TCA metabolites via GC-MS

Bone marrow plasma and peripheral blood plasma samples were spiked in 15 of internal solution containing [^13^C_5_]-labeled analytes for 2-HG, glutamate, and α-ketoglutarate. The proteins were removed by adding 260 μl of chilled methanol and acetonitrile solution to the sample mixture. After drying the supernatant in the speed vacuum, the sample was derivatized with a solution of O-ethylhydroxylamine in pyridine (20 mg/mL) for 1 h at 35 °C, followed by sylation with MtBSTFA + 1% tBDMCS for 1 h at 70 °C before it was analyzed on an Agilent 5977B GC/MS under electron impact and single ion monitoring conditions. Concentrations of α-ketoglutarate (m/z 360.2), 2-HG (m/z 433.2), and glutamate (m/z 432.2) were measured against a 12-point calibration curves that underwent the same derivatization. Finally, validation of the concentrations of glutamate and aspartate in each of the bone marrow plasma samples obtained by the GC-MS methodology was verified by LC-MS.

### Validation of the quantification of glutamate and aspartate by LC-MS

Quantitative analysis of glutamate and aspartate were performed as previously described [34] and summarized as follows. Plasma samples and amino acid calibration standards were prepared with MassTrak Amino Acid Analysis Solution (AAA) kit from Waters according to instructions with slight modifications for detection on a mass spectrometer [34]. A 10-point standard concentration curve was made from the calibration standard solution to calculate the concentrations of glutamate and aspartate in plasma samples [34]. A solution containing the required isotopes of glutamate and aspartate were purchased commercially and used as the internal standard solution [34]. Frozen bone marrow plasma samples were thawed, spiked with internal standard, and then deproteinized with cold MeOH followed by centrifugation at 10,000×*g* for 5 min prior to derivatization with the derivatizing reagent 6-aminoquinolyl-N-hydroxysuccinimidyl carbamate according to MassTrak instructions [34]. High-resolution separation was done using an Acquity UPLC system, injecting 1 μl of derivatized solution, with a UPLC BEH C18 1.7 micron 2.1 × 150 mm column from Waters [34]. Column flow was set to 400 μl/min with a gradient from 99.9%A to 98%B where buffer A is 1% acetonitrile in 0.1% formic acid and buffer B is 100% acetonitrile [34]. A column temp of 43 °C and a sample tray temp of 6 °C was set and mass detection was completed on a TSQ Quantum Ultra from Thermo Finnigan running in positive ESI mode [34]. Finally, the settings of a scan width of 0.002, scan time of 0.04 s per transition mass, collision energy of 25, collision gas pressure of 1.5 mTorr, tube lens value set to 90, and monitoring a signature ion of the derivatized amines at m/z 171.04 by selected reaction monitoring were employed [34]. By using these aforementioned methods, the concentration of aspartate and glutamate were able to be measured.

### Gas chromatography-mass spectrometry (GC/MS)-based isotopomer analyses

Isotopomer analysis of the intracellular and extracellular TCA cycle metabolites from the HMCL cell pellets and spent media, respectively, were performed using an Agilent Technologies 5977B GC/MS (Agilent Inc., CA, USA). Splitless injections of 1 μL aliquots of the derivatized extracts were injected onto a DB5-MS capillary column (30 m × 250 μm i.d., 0.25-μm film thickness; J&W Scientific, Folson, CA). The mass spectrometer (MS) was operated under electron impact (EI) conditions with selected ion monitoring (SIM). The temperature of the injector was set at 250 °C and the transfer line to the MS at 280 °C. Helium was used as the carrier gas at a flow rate of 1 mL/min. The GC temperature program used was oven held for 0.5 min at 120 °C, then increased to 180 °C at 25 °C/min and then to 270 °C at 6 °C/min, and finally increased to 325 °C at 30 °C/min with a hold time of 1 min. Data were processed using MassHunter quantitative analysis software version B.08.00 build 8.0.598.0 (Agilent Technologies Inc., CA, USA) for integration of peaks and calculation isotopic ratios.

SIM was used to monitor the mole percent enrichment for each analyte, such as the fragment (M0) and all labeled mass isotopomer positions (M1, M2, M3 etc.) up to m+2 above the number of carbons in the molecule backbone. M/z values of M0 were monitored for the following intermediates: lactate (m/z 261.2), fumarate (m/z 287.1), succinate (m/z 289.1), α-ketoglutarate (m/z 360.2), malate (m/z 419.3), citrate (m/z 591.4), 2-HG (m/z 433.2), and glutamate (m/z 432.2). The mass isotopomer distribution of each compound was then corrected for natural abundance using the respective standards [35]. We used an appropriate set of linear simultaneous equations to calculate mole percent enrichment of TCA cycle intermediates to understand glucose-dependent or glucose-independent glutamine metabolism in myeloma cells.

### Statistical analysis

Fractional abundance of ^13^C in the various TCA metabolites is expressed as mean ± standard error mean (SEM). Data were compared and analyzed using the paired *t* test, and significance was defined as *p* < 0.05. For the quantitative levels of TCA cycle intermediates, given the non-normal distribution of the data, the differences between groups of interest were compared and analyzed using the non-parametric Mann-Whitney *U* test, and significance was defined as *p* < 0.05. This analysis was performed using GraphPad Prism version 7.00 for Windows, GraphPad Software, La Jolla, CA, USA, www.graphpad.com.

## Supplementary Information

**Additional file 1: Supplementary Figure 1**. Expected mass isotopomer distribution of the intermediates in the first turn of the TCA cycle as a result of glutamine anaplerosis of U-^13^C_5_-Glutamine. Each intermediate is represented by the number of carbon atoms in their molecular structure with green circle representing ^13^C and black circles representing ^12^C. **Supplementary Figure 2**. The percent intracellular isotopomer distribution of metabolites from the first turn of the TCA cycle in RPMI-8226 and MM1S human myeloma cell lines relative to the U-^13^C-glutamine enrichment in the cell culture media. **Supplementary Figure 3.** Relative differences in mRNA expression of SNAT1 and LAT1 between CD138 + cells from MGUS (*N* = 6) and MM (*N* = 10) patients in context of their effect on glutamine transport into the cell. **Supplementary Figure 4.** XY correlations of the absolute concentrations of glutamate and aspartate in the bone marrow plasma of patients with MGUS (*N* = 11) and MM (*N* = 12) utilizing GC-MS vs. LC-MS.

**Additional file 2: Supplementary Table 1.** Comparisons of the levels of mRNA of select genes associated with glutamine metabolism via RNA sequencing between MM and MGUS groups and CD138 +/- cells.

## Data Availability

The datasets used and/or analyzed during the current study are available from the corresponding author on reasonable request.

## Abbreviations

MGUS: Monoclonal gammopathy of undetermined significance
MM: Multiple myeloma
SMM: Smoldering multiple myeloma
TCA: Tricarboxylic acid
cPCs: Clonal plasma cells
GLS: Glutaminase
SIRM: Stable isotope-resolved metabolomics
HMCL: Human myeloma cell line
