# Supplementary material for: In vivo assessment of glutamine anaplerosis into the TCA cycle in human pre-malignant and malignant clonal plasma cells
Source: Cancer Metab. 2020 Dec 11;8:29. doi: 10.1186/s40170-020-00235-4 (PMC7731537; doi:10.1186/s40170-020-00235-4)
Supplement: Supplementary file 1 — Additional file 1: Supplementary Figure 1. Expected mass isotopomer distribution of the intermediates in the first turn of the TCA cycle as a result of glutamine anaplerosis of U-13C5-Glutamine. Each intermediate is represented by the number of carbon atoms in their molecular structure with green circle representing 13C and black circles representing 12C. Supplementary Figure 2. The percent intracellular isotopomer distribution of metabolites from the first turn of the TCA cycle in RPMI-8226 and MM1S human myeloma cell lines relative to the U-13C-glutamine enrichment in the cell culture media. Supplementary Figure 3. Relative differences in mRNA expression of SNAT1 and LAT1 between CD138 + cells from MGUS (N = 6) and MM (N = 10) patients in context of their effect on glutamine transport into the cell. Supplementary Figure 4. XY correlations of the absolute concentrations of glutamate and aspartate in the bone marrow plasma of patients with MGUS (N = 11) and MM (N = 12) utilizing GC-MS vs. LC-MS. [file 40170_2020_235_MOESM1_ESM.pdf]

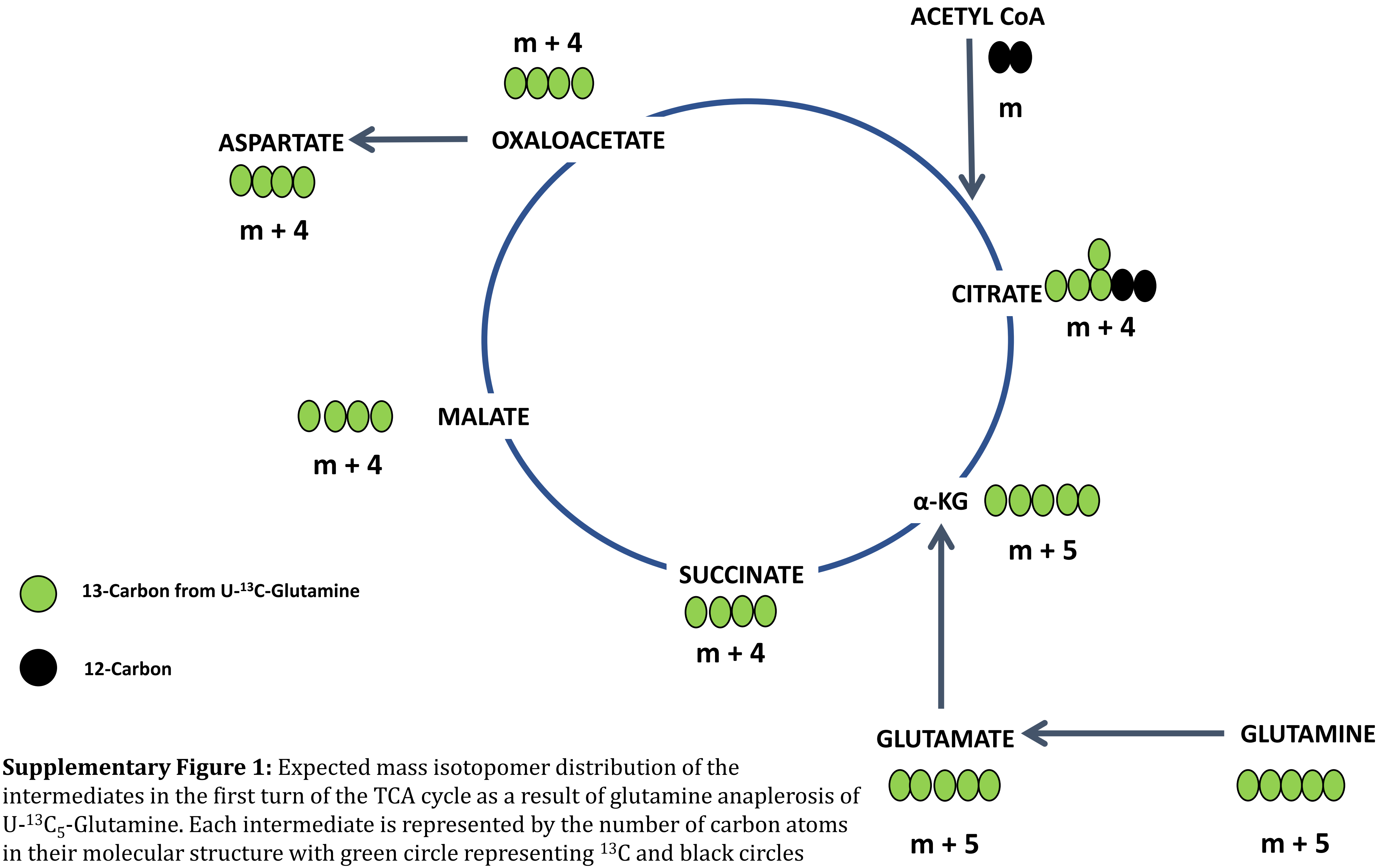

Supplementary Figure 1: Expected mass isotopomer distribution of the intermediates in the first turn of the TCA cycle as a result of glutamine anaplerosis of U-$^{13}C_5$-Glutamine. Each intermediate is represented by the number of carbon atoms in their molecular structure with green circle representing ^{13}C and black circles representing ^{12}C.

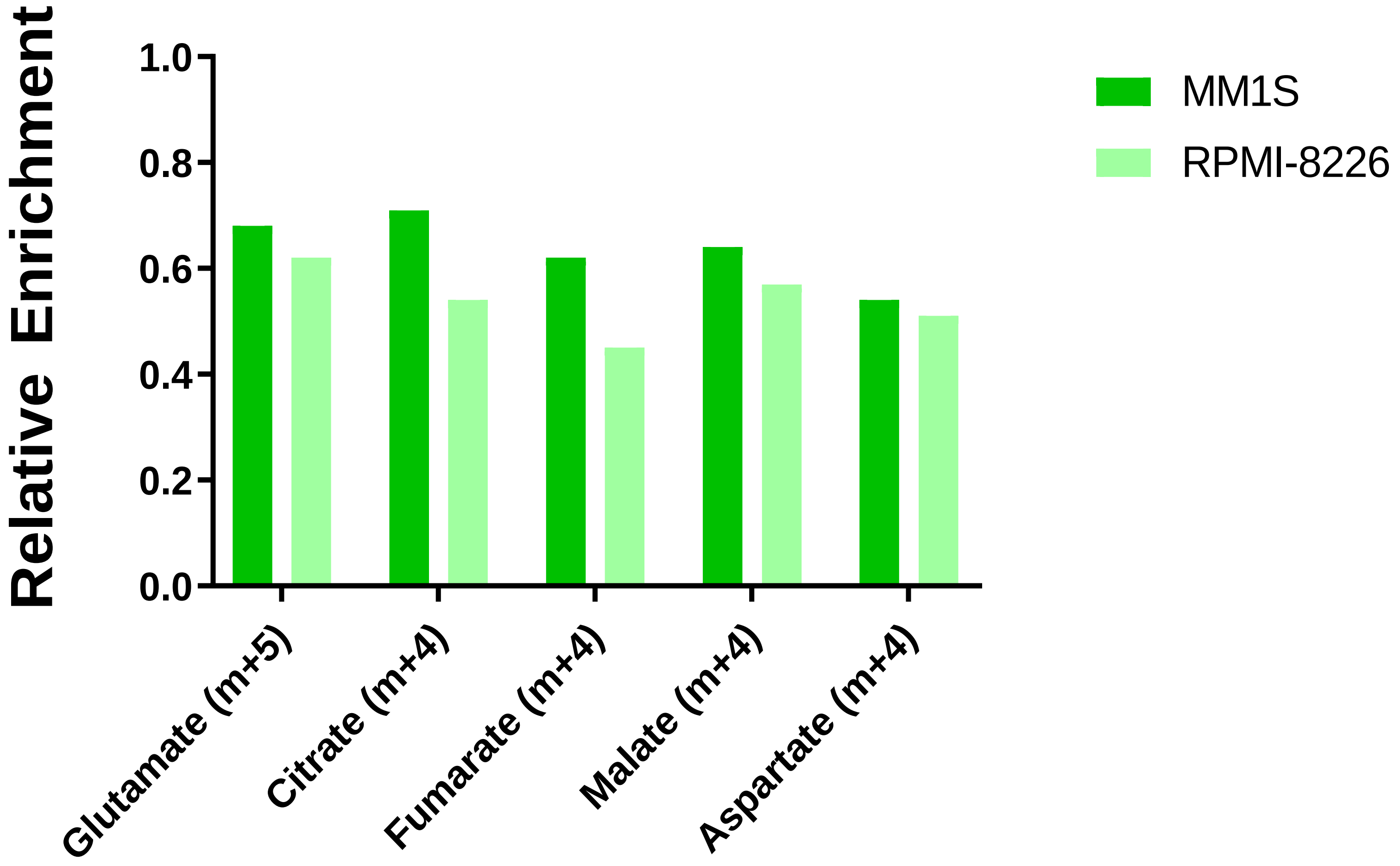

Supplementary Figure 2: The percent intracellular isotopomer distribution of metabolites from the first turn of the TCA cycle in RPMI-8226 and MM1S human myeloma cell lines relative to the U-^{13}C-glutamine enrichment in the cell culture media.

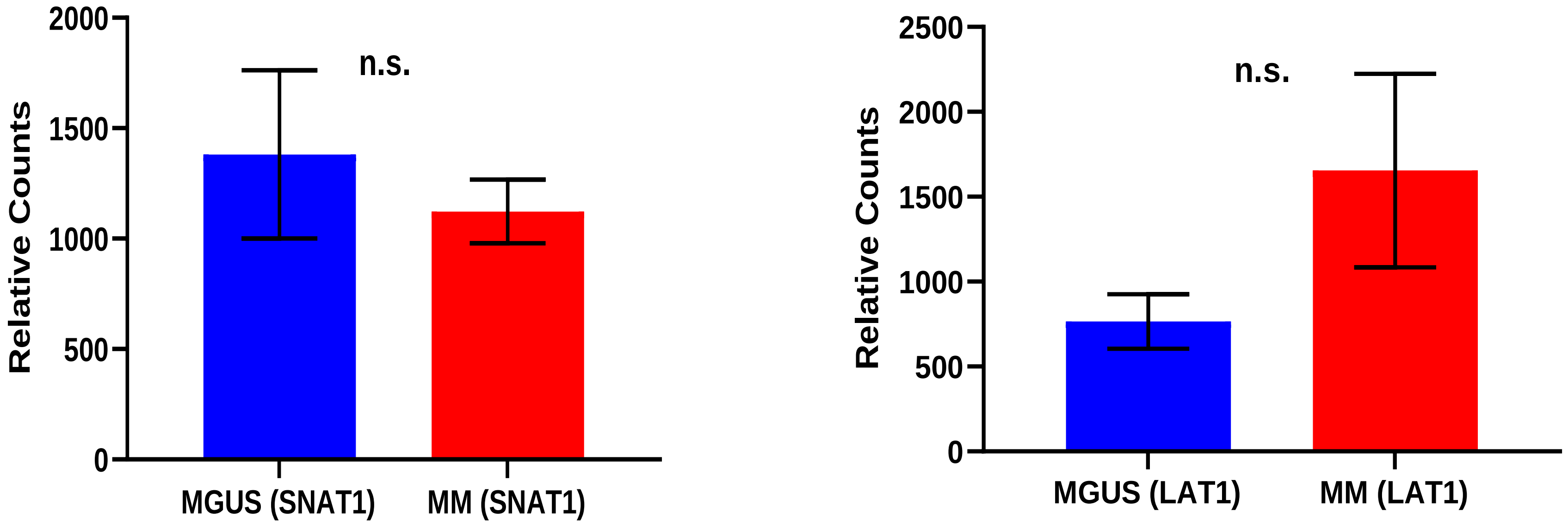

Supplementary Figure 3: Relative differences in mRNA expression of SNAT1 and LAT1 between CD138 + cells from MGUS (N = 6) and MM (N = 10) patients in context of their effect on glutamine transport into the cell.

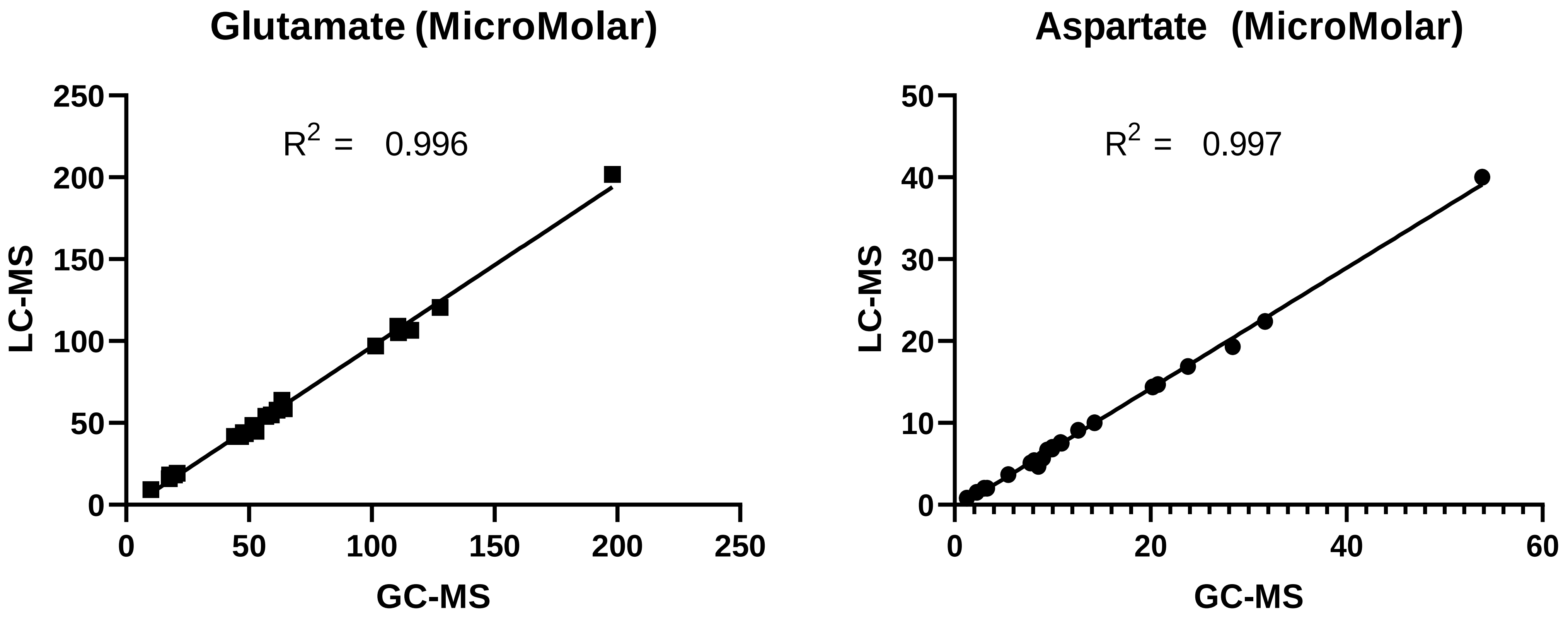

Supplementary Figure 4: XY correlations of the absolute concentrations of glutamate and aspartate in the bone marrow plasma of patients with MGUS (N = 11) and MM (N = 12) utilizing GC-MS vs. LC-MS.
