# Supplementary material for: In vivo assessment of glutamine anaplerosis into the TCA cycle in human pre-malignant and malignant clonal plasma cells
Source: Cancer Metab. 2020 Dec 11;8:29. doi: 10.1186/s40170-020-00235-4 (PMC7731537; doi:10.1186/s40170-020-00235-4)
Supplement: Supplementary file 2 — Additional file 2: Supplementary Table 1. Comparisons of the levels of mRNA of select genes associated with glutamine metabolism via RNA sequencing between MM and MGUS groups and CD138 +/- cells. [file 40170_2020_235_MOESM2_ESM.zip › supptable1.pdf]

Supplementary Table 1: Comparisons of the levels of mRNA of select genes associated with glutamine metabolism via RNA sequencing between MM and MGUS groups and CD138 +/- cells.

GENE	P-value	FDR step up	Fold change	Comparison groups
**MYC**	0.00000	0.00000	9.5	MM (138+ cells vs 138- cells)
**MYC**	0.00339	0.01224	5.6	MGUS (138+ cells vs 138- cells)
**MYC**	0.00205	0.01556	3.2	CD138+ cells (MM vs MGUS)
**GLS**	0.00005	0.00023	2.4	MM (138+ cells vs 138- cells)
**GLS**	0.00109	0.00517	2.6	MGUS (138+ cells vs 138- cells)
**GLS**	0.54622	0.71730	1.1	CD138+ cells (MM vs MGUS)
**SLC1A5 (ASCT2)**	0.00066	0.00671	2.7	MM (138+ cells vs 138- cells)
**SLC1A5 (ASCT2)**	0.00641	0.02013	4.3	MGUS (138+ cells vs 138- cells)
**SLC1A5 (ASCT2)**	0.00000	0.00000	9.2	CD138+ cells (MM vs MGUS)
**SLC38A5 (SN2)**	0.00000	0.00005	4.5	MM (138+ cells vs 138- cells)
**SLC38A5 (SN2)**	0.06395	0.12489	2.8	MGUS (138+ cells vs 138- cells)
**SLC38A5 (SN2)**	0.00000	0.00000	10.6	CD138+ cells (MM vs MGUS)
